# Antifungal Activity of Brilacidin, a Nonpeptide Host Defense Molecule

**DOI:** 10.3390/antibiotics13050405

**Published:** 2024-04-28

**Authors:** David J. Larwood, David A. Stevens

**Affiliations:** 1 Department of Pharmaceutical Chemistry, University of California-San Francisco, San Francisco, CA 94158, USA; david.larwood@ucsf.edu; 2 California Institute for Medical Research, San Jose, CA 95128, USA; 3 Valley Fever Solutions, Tucson, AZ 85719, USA; 4 Division of Infectious Diseases and Geographic Medicine, Stanford University Medical School, Stanford, CA 94305, USA

**Keywords:** brilacidin, synthetic nonpeptide mimetics, antifungal activity, defensins, antimicrobial peptides, AMP

## Abstract

Natural host defensins, also sometimes termed antimicrobial peptides, are evolutionarily conserved. They have been studied as antimicrobials, but some pharmaceutical properties, undesirable for clinical use, have led to the development of synthetic molecules with constructed peptide arrangements and/or peptides not found in nature. The leading development currently is synthetic small-molecule nonpeptide mimetics, whose physical properties capture the characteristics of the natural molecules and share their biological attributes. We studied brilacidin, an arylamide of this type, for its activity in vitro against fungi (40 clinical isolates, 20 species) that the World Health Organization has highlighted as problem human pathogens. We found antifungal activity at low concentrations for many pathogens, which indicates that further screening for activity, particularly in vivo, is justified to evaluate this compound, and other mimetics, as attractive leads for the development of effective antifungal agents.

## 1. Introduction

Peptide antibiotics (e.g., vancomycin, daptomycin, polymyxin, echinocandins) have shown their value in clinical medicine. There are >2000 discovered natural “antimicrobial peptides” (AMPs) which are highly evolutionarily conserved and present in microbes, plants, and all vertebrates [1,2]; >100 are known to be produced by humans [3,4]. A broad antimicrobial spectrum is a group characteristic, most are amphiphilic and cationic [3]. These peptides are better termed “host defense peptides” or defensins, because they are part of the host’s innate immune response and are the first line of defense [5,6]. Many of these appear to have broad biological functions, as will be further discussed.

There has been longstanding interest in exploiting such molecules, and their analogues, as clinical anti-infectives, with stimulation to expand our armamentarium owing to the development of resistance to current chemically synthesized molecules and other natural products. Natural AMPs may be undesirable for clinical therapeutics because of instability, degradation by host proteases, low solubility, reduced activity in the presence of salts or DNA, short half-lives in vivo, difficult and expensive manufacturing issues, and the possibility of the development of antibodies in heterologous hosts [6,7,8,9,10,11]. This led to the development of synthetic AMPs, using amino acid sequences and/or amino acids not found in nature, which ameliorated some of these problems [12,13]. It was then discovered that the physicochemical properties of the synthetic molecules were more important than the sequence of the amino acids [10,11,14], and, with attention to the secondary structure, charge, and folding, that totally synthetic non-peptide molecules could recapitulate the structural properties of AMPs and mimic their activities [10,11]. A lead candidate from this line of research is brilacidin, a cationic water-soluble amphiphilic helical arylamide with discrete nonpolar hydrophobic and polar hydrophilic regions and a polymer backbone [10]. The present study is an initial exploration of the antifungal spectrum of brilacidin, with particular attention to pathogens for which there is a huge present clinical burden (e.g., cryptococcosis in Africa in the wake of the AIDS epidemic) and those pathogens for which present clinically available antifungals provide insufficient efficacy.

## 2. Results

The screening of the selected fungal pathogens of great interest is displayed in Table 1. The low MIC values (largely < 4 µg/mL) of all in this group, except *A. fumigatus*, suggests that brilacidin is worthy of study in animal models to ascertain whether this level of potency in vitro will translate into efficacy in vivo and thus have potential clinical utility. These MIC values in µg/mL are favorable compared to those of conventional antifungals.

There is a disparity between this 50% inhibition and the elevated 100% inhibition MICs for *Coccidioides*, *Mucorales*, *Sporothrix*, and *Fusarium*, suggesting that for those pathogens, brilacidin’s antimicrobial activity is unlike that of polyenes. Polyenes, such as amphotericin B, typically have similar concentrations for 50% and 100% inhibition and even for cidal activity [15]. However, the clinical utility of azoles and echinocandins, which also do not produce even 100% fungal inhibition in vitro, suggests that conclusions about the efficacy of brilacidin in vivo need to be deferred until animal models are explored. The most striking, consistent results are those against *C. neoformans*, where brilacidin appears to have unique antifungal activity among these pathogens assayed.

The studies displayed in Table 2 represent an initial screening effort to examine whether other groups of pathogens may be worthy of the broader screening displayed in Table 1. Several of these pathogens are in the favorable range discussed for pathogens studied as per Table 1 and should be more extensively screened in the future; the initial results with *Nakaseomyces glabratus* and *Candida auris* do not as yet, unfortunately, give such indication.

## 3. Materials and Methods

### 3.1. Drugs

Brilacidin (N4, N6-bis(3-(5-gaunidinopentanamido)-2-(R)-pyrrolidin-3-yl)oxy)-5-(trifluoromethyl)phenyl)pyrimidine-4,6-dicarboxamide tetrahydrochloride) (C40H50F6N14O6.4HCl), MW 1082.7, sterile and >98% pure, was supplied by Innovation Pharmaceuticals, Wakefield, MA, USA. It was supplied as a solid and was readily soluble in water and liquid media, such as RPMI-1640. To convert µg/mL, as expressed in this paper, to millimolar, multiply µg/mL by 0.924. 

In prior studies for some isolates, as mentioned, azoles were supplied by Pfizer Inc., Groton, CT, USA; echinocandins by Merck, Inc., Rahway, NJ, USA; and amphotericin B by the Bristol-Myers Squibb Company, Princeton, NJ, USA. 

### 3.2. Isolates

The World Health Organization has recently identified particular fungal pathogens as needing attention because of epidemiological reasons and/or resistance to many available drugs [16]. It was this document that guided our selection of isolates, constrained by the availability of isolates in our collections. The isolates were all recent clinical isolates, sent to our laboratories for clinical testing, with three exceptions (CN9759, Silv., 10AF), which were originally clinical isolates but were maintained in the laboratory because they have desirable characteristics for animal model studies, which may be indicated in the future. All were tested using their CIMR accession numbers, without any patient identification.

### 3.3. Testing

Testing was performed by standard broth dilution methods detailed elsewhere [17,18,19]. The RPMI-1640 medium is desirable because it is fully defined and it also allows microbial susceptibility testing in the presence of mammalian cells in the future. Testing of *Coccidioides* was performed under BSL3 conditions. A stock solution was made of 640 µg/mL. The range of concentrations tested, in 2-fold dilutions, was 0.5–64 µg/mL. For the testing of a new drug, it is not clear whether a 50% inhibition endpoint for yeasts (equivalent to a Minimum Effective Concentration, that concentration producing a morphological change in filamentous fungi), as is used clinically for azoles and flucytosine, or a 100% inhibition endpoint (i.e., a tube as clear as the starting inoculum), as is used clinically for polyenes, is most relevant, so both endpoints were determined for brilacidin. In isolated instances where relevant (mentioned in the tables) azole resistance was defined as 50% inhibition at ≥64 µg/mL, echinocandin resistance as 50% inhibition at ≥3.1 µg/mL, and amphotericin intermediate as 100% inhibition at ≥2 µg/mL. Testing was repeated in approximately 20% of the assays and was always reproducible. Every assay included a positive concurrent control, embodying a pan-susceptible *Candida kefyr*, and fluconazole (MIC < 0.5 µg/mL).

## 4. Discussion

The activities of AMPs have been described against bacteria, protozoa, and viruses [2,20,21,22]. Several theoretical models exist to explain their interactions with cells [2,22,23]. The antifungal activity of other AMPs and their analogues has previously been demonstrated [3,4,12,13,24,25,26,27,28], including, in our prior study, against pathogens resistant to specific antifungals [13] and with cidal activity sometimes demonstrated [13,27]. A topically applied AMP has already shown antifungal efficacy in patients [23]. In the present study, conidia or yeasts were used as the inoculum. The conidia develop during the assay to hyphae; thus, in the case of filamentous organisms, antifungal activity against conidia themselves, during transformation to hyphae or on hyphal development, could produce positive test results. Prior studies have indicated AMP activity against all these phases [4,29]. Our results, with our testing methods, are consistent with the observed rapid antifungal action of AMPs [13,24]. The present study shows brilacidin activity in vitro against several problem fungal pathogens. For possible clinical interest, these studies must be expanded to further study brilacidin’s pharmacology, tissue penetration, and toxicology. What is not yet understood is why there are the species differences in susceptibility that we have demonstrated, and this may relate to differences in susceptibility to the mechanism(s) of drug action. More studies, with other fungal species, are required. Although brilacidin has been shown to depolarize the *A. fumigatus* cell membrane and to disrupt the cell wall [30], our results (minimal activity against this genus) present a difference from the inhibitory activity against *A. fumigatus* demonstrated for some AMPs [4]. A caution regarding this subject is that some AMPs have also been shown to increase *A. fumigatus* growth in vitro [4,27]. 

Prior studies have indicated the synergy in vitro of AMPs and their analogues with conventional antimicrobials and antifungals [7,8,26,28,30], even with host AMPs [8], which is an avenue for further exploration. One possible mechanism for any such synergy is that AMP increases the permeability of, and depolarization of, the pathogen membrane, allowing greater penetration of the conventional drug [6,31,32]. Brilacidin synergy with an antifungal in vivo has been shown [30].

It is unclear what in vitro test characteristics, aside from whether to use 50% or 100% endpoints, will be most useful to predict activity in vivo. Which medium is the best needs determination, as well as the conditions of pH, ionic concentration, oxygenation, and buffer [29]. It may be most relevant to study these agents in the presence of host cells, and, depending on the target in vivo, to test in a milieu that reflects the tissue situation, such as artificial sputum medium, as we have done [33]. Testing against fungal biofilms may be more relevant than against planktonic growth for many clinical situations [34], and AMPs have been demonstrated to inhibit biofilms [1,8,13,26,35].

Mechanisms of action for AMPs and their analogues include: insertion into pathogen (and host) membranes (with creation of pores) or other phospholipids and/or into ribosomal subunits, stress on protein folding, stress of cell membranes, increase of reactive oxygen species; affecting intracellular calcium concentrations, affecting the proteome, inactivation of cellular proteins; affecting cell signaling, the regulation of cell death, binding the anionic nucleic acids and/or affecting their synthesis, preventing biofilm formation, regulating iron metabolism, the inhibition of cellular enzymes, the activation of cell wall lytic enzymes, binding of glucan and/or chitin, the modulation of the cell wall to expose beta glucan, and the degradation of cell walls [1,2,3,4,6,7,8,12,23,25,27,28,36,37]. 

Given AMP’s effects on the regulation of many genes in their targets [6] and all these possible mechanisms of action, many effects on host function have also been described for them, including affecting host cell differentiation, immunomodulation, the regulation of cytokines, opsonization, the regulation of inflammation, the increase of phagocytosis, the stimulation of chemotaxis (for neutrophils, monocytes, and lymphocytes), the activation of eosinophils and angiogenesis, and the activation of epithelial cells [1,2,3,7,27,38]. It is likely that these possible host effects would come into play if brilacidin were to be used as an antifungal in vivo, and this may make MIC’s absolute values, or differences, in vitro less important for the effect on the outcome.

The development of resistance to AMPs has been shown generally difficult for microbes to achieve [6], and that has been corroborated for peptide AMPs [4], synthetic peptides [13], and brilacidin [10]. AMP action on several different microbial processes, as detailed above, may explain AMP’s breadth of microbial spectrum [3], as shown in our results here with various species, and AMP’s defense against resistance development [1]. Previous observations of the development of resistance to AMPs have included the development of microbial efflux pumps, which may be lessened for the nonpeptide mimetics [8]. The cationic nature of brilacidin and its water solubility may relate to its ability to target charged fungal membranes [2,11]. Brilacidin depolarization of microbial membranes and its induction of membrane and cell wall stress have been demonstrated [10]. 

The structure of the nonpeptide mimetics preserves the AMP theme of such biologically active molecules having both a charged face and a hydrophobic face [6,39]. The activity of these mimetics is more closely linked to their physicochemical properties than the details of the structures [40]. This nature of this class of molecules allows for studies of molecular modifications that could improve efficacy and decrease undesirable effects [12]. Its manipulation of charge, amphiphilicity, hydrophobic–hydrophilic balance, and folding properties create possibilities for the future. Presently, brilacidin is being studied in human clinical trials for other indications and is not yet focused on fungal infections.

## Figures and Tables

**Table 1:** Brilacidin activity against problem pathogens.

Brilacidin MICs			
Pathogen	Strain	50% Inhibition	100% Inhibition
*Coccidioides posadasii*	Silv.	4	>64
*Coccidioides* sp.	22-50	2	>64
	22-40	2	>64
	22-35	2	>64
	22-33	2	>64
*Aspergillus fumigatus*	18-31	>64	>64
	13-130	>64	>64
	19-12	>64	>64
	21-23	64	>64
	09-03	>64	>64
	18-32	64	>64
	18-117	>64	>64
	13-30	>64	>64
	11-13	>64	>64
	09-117	>64	>64
	10AF	64	>64
*Aspergillus lentulus* (voriconazole resistant)	14-39	32	>64
*Aspergillus terreus*	12-70	>64	>64
*Aspergillus niger*	22-4	8	16
*Lomentospora prolificans*	15-101	4	8
	15-99	4	8
	15-97	4	8
	15-98	4	8
	94-58	8	16
	10-03	4	8
	15-100	8	16
*Scedosporium apiospermum* *complex*	12-13	4	8
	98-38	2	8
(*Scedosporium apiospermum* …)	01-48	4	16
	10-23	2	4
	18-46	8	16
*Fusarium species*	07-144	4	16
	22-51	8	16
	07-136	2	16
	00-137	2	32
	19-171	2	32
	12-22	1	64
	22-1	2	32
*Mucorales*			
*Rhizopus species*	16-88	4	16
	20-235	16	32
	21-01	8	16
	13-91	2	8
	94-2	2	32
	21-85	4	64
*Mucor species*	20-177	16	32
	15-64	4	64
	13-39	4	32
	13-127	4	>64
*Unspeciated zygomycete*	07-140	2	16
*Sporothrix brasiliensis*	20-18	8	64
	20-19	16	64
	20-20	16	64
*Sporothrix schenckii*	20-45	4	16
	20-46	8	32
*Cryptococcus neoformans*	00-288	1	2
	01-126	1	1
	06-71	1	1
	00-289	1	2
	97-370	2	2
	CN9759	1	8
	17-66	2	2

**Table 2:** Initial screen of Brilacidin activity against other problem pathogens.

Brilacidin MICs			
Pathogen	Strain	50% Inhibition	100% Inhibition
*Candida albicans*	20-132	1	4
	5	4	>64
(*C. albicans,* fluconazole-resistant)	21-76	32	>64
*Candida auris*	20-253	>64	>64
*Candida krusei* (fluconazole-resistant)	03-287	8	16
*Candida lusitaniae* (amphotericin-intermediate)	22-16	8	8
*Torulopsis glabrata* (*Nakaseomyces glabratus*)	22-21	64	>64
*Acremonium species* (resistant to azoles, polyenes, echinocandins)	18-51	4	>64
*Exserohilum species*	19-48	1	16

## Data Availability

Tables of the original raw data are available from the corresponding author.
